# Novel Nanofluidic Cells Based on Nanowires and Nanotubes for Advanced Chemical and Bio-Sensing Applications

**DOI:** 10.3390/nano11010090

**Published:** 2021-01-03

**Authors:** Xin-Yi Zhu, Bo-Ran Wang, Yi Gu, Hao Zhu, Lin Chen, Qing-Qing Sun

**Affiliations:** The State Key Laboratory of ASIC and System, School of Microelectronics, Fudan University, Shanghai 200433, China; 19112020059@fudan.edu.cn (X.-Y.Z.); 20112020032@fudan.edu.cn (B.-R.W.); 20112020008@fudan.edu.cn (Y.G.); linchen@fudan.edu.cn (L.C.); qqsun@fudan.edu.cn (Q.-Q.S.)

**Keywords:** nanowire, nanotube, nanofluidic cell, single molecule detection, chemical and bio-sensing

## Abstract

Since the first introduction of one-dimensional nanochannels for single-molecule detection, there has been increasing interest in modern nanofluidic systems, such as chemical and biological sensing applications. Recently developed nanowires (NWs) and nanotubes (NTs) have received tremendous attention due to their unique geometrical, physical and chemical properties, which are very attractive in this field. Here, we review the recent research activities in the field of novel nanofluidic cells based on NWs and NTs. First, we give a brief introduction of this field. Then the common synthesis methods of NWs and NTs are summarized. After that, we discuss the working principle and sensing mechanism of nanofluidic devices, which is fundamental to the interaction between these nanostructures and small molecules. Finally, we present the NW- and NT-based devices for chemical and bio-sensing applications, such as gas sensing, pathogen detection, DNA sequencing, and so forth.

## 1. Introduction

Nanofluids are suspensions of basic liquids and nanoparticles, which provide a well transport environment for various ions, gases and bio-molecules. The development of nanofluidics has led to the preparation and optimization of many new engineering devices for biochemical sensor applications, such as gas sensing, protein and pathogen detection, DNA translocation and sequencing, etc. [1]. It was realized that the electronic devices should be further scaled down to the size of chemical molecules with enhanced performance to achieve biological applications. The emergence of various types of nanomaterial has enabled the preparation and application of nanochannels with adjustable structures and shapes [2,3]. In 1996, Kasianowicz et al. first reported the translocation of DNA through a α-hemolysin protein nanopore under an external electrical field [4]. Since then, other nanostructures have been extensively studied and provide a new platform for nanofluidic system. For example, the research on two-dimensional (2D) materials (graphene, MoS_2_, BN, etc.) has greatly enriched and promoted the field of nanofluid ion transport [5]. Compared with nanopores or other nanofluidic channels, 2D nanofluidic channel systems with high flow rates are relatively easy and expandable to manufacture, which can be used for applications in bionic transmission and manipulation of ions, molecular screening, and energy conversion [6,7,8]. However, the currently reported research on the asymmetric ion transmission characteristics of the 2D nanofluidic homogeneous structure is still in its infancy, due to their problems of insufficient charge density and low rectification ratio [9].

Since carbon nanotubes (CNTs) were first synthesized, tremendous efforts have been made in the research field of one-dimensional (1D) materials, including the synthesis, properties, assembly and application [10]. So far, there has been significant research devoted to various solid nanowires and tubular forms, such as carbon, silicon, boron nitride, oxides (SiO_2_, Al_2_O_3_, MoO_3_, V_2_O_5_, TiO_2_), chalcogenides (WS_2_, MoS_2_), and halides (NiCl_2_). Nanowires (NWs) and nanotubes (NTs) are seamless cylinders with high aspect ratios, and their structures and compositions lead to unique geometric, physical, and chemical properties. Taking advantage of their controllable diameters, high mechanical strength, high transmission efficiency, and easy planar integration, it has a potential to break through the limitations of traditional nanofluidic applications based on vertical nanoporous films and 2D nanomaterials [11]. From the basic physics research point of view, NTs and NWs provide an interesting system to test classical theory of nanofluid flow. On the other hand, they can be readily integrated into novel nanofluidic devices, which are further explored in single-molecule technique and future bio-chemical sensing applications. NW/NT-based nanofluidic cells sensitively record the nanoparticles and can effectively overcome existing challenges in conventional biological and chemical electronics. For example, a recognition probe fixed to the surface of the nanostructure selectively interacts with target analytes in solution, responding an electrical signal that reflects the concentration. In addition, ions or small molecules with charges can cause physical blockages of nanoscale channels during transport, which results in the measurable and identifiable electrical properties changes.

The core components of these nanofluidic devices are the quasi-1D nanostructures. First, NWs and NTs have the advantages of small geometrical size and high surface-to-volume ratio. This makes it possible to generate obvious electrical signals at ultralow concentrations, allowing the reduction of sample size and the miniaturization of sensors. Secondly, high quality NWs and NTs with uniform inner diameters, robustness, and non-porous smooth surfaces are attractive in the fields of nanoelectronics, optoelectronics, and biochemical sensing. Moreover, the well-defined internal and external surface as well as the sites at the ends can be easily modified, which is useful for ion and molecular detection, capture and manipulation.

This review is organized to emphasize the recent advances in novel nanofluidic cells based on NWs and NTs for advanced chemical and bio-sensing applications, updating the reports of previous works. We will briefly go through the synthesis methods of NWs and NTs in Section 2 and discuss the principles and sensing mechanisms of NW/NT-based nanofluidic devices in Section 3. Several representative advances in nanostructure-based nanofluidic biochemical sensors will be reviewed in Section 4. Based on the fact that there have been some excellent reviews for the research on NWs/NTs-based sensors [12,13,14], we also discussed about novel preparation methods, functional modifications, monitoring mechanisms, and correction schemes, which is of great significance to improve the sensing performance and overcome the limitations of biochemical sensors based on NWs/NTs nanofluidic cells. It should be noted that we will focus on the basic and latest progress in materials preparation, device mechanisms and applications, instead of covering all the work performed in this field.

## 2. Synthesis Approaches

There are two routes in realizing one-dimensional nanomaterials: top-down and bottom-up approaches. Bottom-up is a commonly used approach to synthesize the target structure at atomic or molecular level which makes it feasible to build new conceptual devices and functional systems. The early synthesis approaches of NWs and NTs are mainly achieved by vapor phase growth. In typical bottom-up processes, two basic steps of nucleation and growth are involved to form a solid crystal from a gas, liquid or solid phase. When the concentration of the growth unit is sufficiently high up to saturation, it will aggregate into small crystal nuclei through homogeneous nucleation and continue to grow into larger structures. The robust NWs/NTs and rationally designed nanofluidic cells have advantages in potential nanoelectronics, optoelectronics, and bio-chemical sensing applications. On the other hand, when developing a synthetic method of nanomaterials, attention should be paid to control the morphology, size, composition and uniformity of the products. This section reviews the synthesis approaches of NWs and NTs that have been commonly used and developed in recent years, such as vapor-liquid-solid (VLS) growth, membrane-templet synthesis and nanowire template approach.

### 2.1. Vapor-Liquid-Solid (VLS) Growth

**Figure 1:**
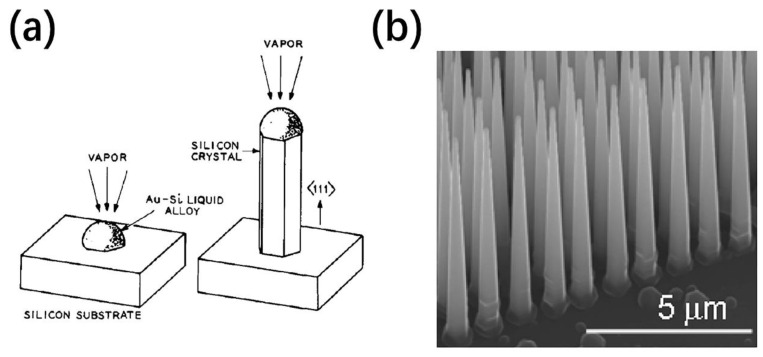
(**a**) Schematic illustration of the vapor-liquid-solid (VLS) growth method. Silicon crystal grows with liquid alloy at the tip [15]; (**b**) scanning electron microscope (SEM) image of SiNWs (nanowires) with significant tapering [25].

Nanoparticle-catalyzed VLS growth is a widely used method for preparing NWs and NTs by gas phase reaction because of its simplicity and versatility. It was first proposed by Wagner in 1964 through the growth of microscopic Si whiskers [15]. Later, the research teams from Harvard University and Hong Kong University proposed the true nanoscale Si NWs [16]. Various single crystal forms of inorganic nanowires have been reasonably synthesized by VLS method with diameters on the order of nanometers [17,18]. The growth mechanism is shown in Figure 1a [15]. Generally, small droplets of metal catalyst (e.g., Au nanoparticles) are assembled or patterned on the solid substrate. Then, the carrier gas stream containing precursors is dissolved at elevated temperature to form alloy droplets. Once the reactants reach supersaturation, they will precipitate and form a solid-liquid interface, which will promote the anisotropic growth of crystals. Thereby the material preferentially grows in one direction and obtain one-dimensional nanostructure. Following the initial VLS mechanism studies, it has been extended to chemical vapor deposition (CVD) method [19,20,21], which can control the reactant sources, growth temperature and so on well during the synthesis process.

As for diameter of 1D nanostructures, it is typically proportional to the size of the catalyst since the alloy droplet used as a soft template is always at the tip. In addition, the average diameter of NWs depends on the growth temperature, growth time, gold layer thickness, flow rate, and so forth [22,23]. Terasako et al. have reported successful suppression over the enhancement of the average diameter as the growth temperature increases utilizing the alternate source supply technique [24]. Similarly, tapering of VLS grown NWs is a common phenomenon resulting from dynamics of the liquid droplet during growth and direct vs. growth on the sidewall (Figure 1b) [25]. Küpers et al. developed a novel two-step approach to realize an untapered morphology with high vertical yield [26]. NW diameters at the top and bottom gradually approach the same with the increasing of V/III flux ratios during the second growth step. The flux dependence of radial vs. growth was theoretically studied and the diffusion on the NW sidewalls was combined into the model to understand the tapering phenomenon. Dejarld et al. also studied the doping effect on the diameter of GaAs NW [27]. Be-doping was found to significantly reduce the diameter and increase the aspect ratio to 50:1, which greatly improves the performance of the optoelectronic devices.

VLS growth allows NWs/NTs to be selectively grown at ordered positions of the substrate patterned by the catalyst, which reduces the introduction of impurities and pollution in the subsequent dispersion and arrangement [28,29,30,31]. Moreover, growth orientations control is also highly desirable. It was found that the preferred growth direction is the one that minimizes the total free energy and can be influenced by diameters [32]. Recently, Wang et al. first demonstrated the surface-parallel growth of SiO_2_ NWs by controlling mass transport [33]. The directional growth of SiO_2_ NWs towards low SiO concentration was obtained at the position with high SiO concentration gradient. The incorporation of metallic catalyst in the synthetic NWs/NTs is another inevitable issue to be addressed which can introduce unwanted doping or impurities in the nanostructures. Floating catalysts (Mg–Fe–O species) and remote catalyzation methods have the potential to produce high purity BN NTs without post-treatment [34]. Finally, additional treatments are required to modify the electrical properties before assembly into suitable device structures and applications, which will be covered in the following sections.

### 2.2. Membrane-Template Synthesis

Membrane-template synthesis is a simple, low-cost approach for the preparation and assembly of 1D nanomaterials, which refers to copying the specific structure of a template into the product. A porous membrane is one of the basic hard templates, such as anodic aluminum oxide (AAO) membrane, track-etched polycarbonate (PC) membrane, and mesoporous silica. Among them, AAO membranes with higher porosity are usually used to prepare highly periodic arrays [35,36]. PC membranes are commonly available in a variety of pore sizes and larger dimensions, which is more suitable for biosensing applications [37]. In this method, the growth of nanomaterials was guided through nanopores of the membranes, using vapor deposition, electrodeposition, electrochemical deposition and other filling processes to synthesize desired materials. After that, the template can decompose and dissolve through the solvent or annealing.

**Figure 2:**
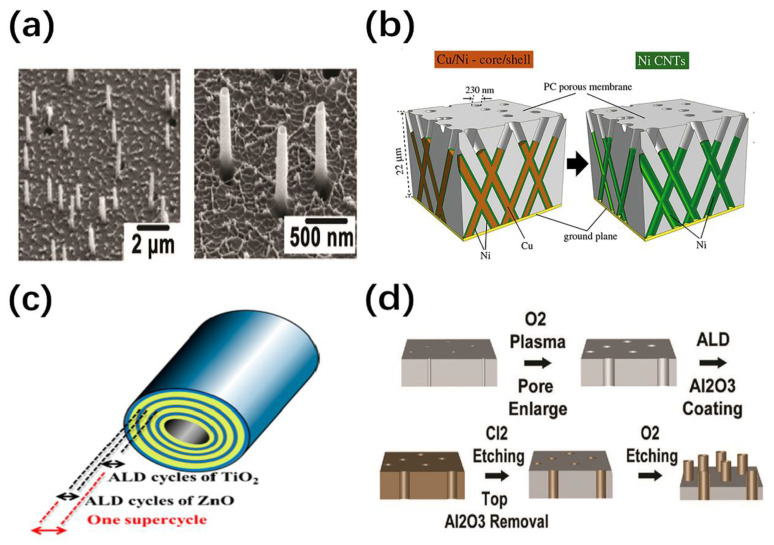
(**a**) SEM image of nanowires array [40]; (**b**) schematic illustration of 3-D Cu/Ni core/shell and Ni carbon nanotubes (CNTs) [41]; (**c**) schematic diagram of highly homogeneous Al- and Zn-doped TiO_2_ nanotube by ALD with various cyclic sequences [43]; (**d**) schematic illustration of fabrication of nanostraws array using a track-etched polycarbonate membrane [40].

Membrane-template synthesis has the characteristics of high periodicity, chemical stability and versatility. By controlling the diameter, spacing, and direction of the pores, a uniform, dense, and nearly parallel 1D nanomaterial matrix can easily be obtained (Figure 2a) [38,39,40]. The solid or hollow cylinders depend on the hole wall chemistry, deposition time and deposition method. Moreover, Three-dimensional NWs or NTs networks with precise spatial distribution, diameter and wall thickness have been prepared (Figure 2b) [41,42]. The improved method has been developed to synthesis AL-, Zn- doped TiO_2_ nanotubes with uniform continuous composition control using PC membrane and atomic layer deposition (ALD) (Figure 2c) [43]. Another reproducible method is using alumina nano-trenches with single-wall NTs (SWNTs) as templates. CNTs were removed by O_2_ reactive ion etching (RIE) following the area-selective ALD of alumina coating, which obtained predefined alumina membrane with nano-trenches that used as a mask to obtain sub-5 nm ZnO nanowires [44].

Despite the above characteristics, the membrane-template approach also has some intrinsic limitations. It becomes difficult to manipulate without order after being released from the membrane. There are two methods for producing ordered arrays onto the target substrate. The first is to prepare the template directly on the substrate, but the film thickness may be limited [45,46]. The other method involves the transfer method, which is complicated to operate and the membrane is fragile. An improved transfer method that allows the integration of dense NW arrays on almost any flat and curved substrate has been demonstrated [47]. A two-layer polymer (S1818 and PMMA A11) was used to prevent damage to the AAO membrane and the residue of organic contamination in pores. Secondly, the custom-designed nanopores membranes are very expensive or commercially unavailable. He et al. produced the membranes with desired nanopore size by oxygen plasma etching for the application of cell penetration and DNA infection (Figure 2d) [40]. The composite fiber melt-codrawing and bundling method was developed to prepared a well-controlled porous PMMA membrane with controlled structure size [39]. Unfortunately, the membrane-template approach relies on wet chemical reactions at low temperatures, resulting in a lack of layered crystal structures, poor morphology and porous walls.

### 2.3. Nanowire Template Approach

A promising approach that uses nanowires as epitaxial growth substrates or sacrificial templates has been developed in recent years. In this approach, nanowires are converted into core-sheath structures through deposition or chemical methods and etch the cores subsequently [48]. There are various nanowires with different composition and size that can be used as stable templates. In addition, advanced conformal coating technologies such as CVD, ALD and sputtering allow epitaxial growth of various high-quality materials. The internal core can be selectively removed based on different chemical reactivities.

**Figure 3:**
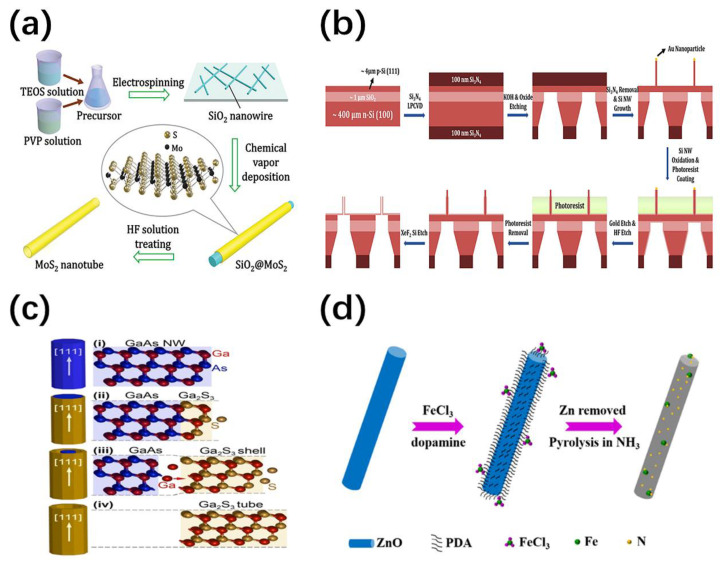
(**a**) Schematic illustration of MoS_2_ nanotube synthesis [49]; (**b**) schematic illustration of preparation of SiO_2_ nanotubes from Si nanowires on a SOI substrate [52]; (**c**) reaction schematic of the conversion of GaAs nanowires into single-crystalline γ-Ga_2_S_3_ nanotubes [53]; (**d**) schematic illustration of the fabrication of FeN_x_ modified CNT using ZnO nanowire template [55].

The NTs prepared by nanowire template approach have the smooth surface and lower porosity. The diameters can be tuned from 1 to 100 nm and the inner walls can easily covalently attach the desired receptors, which is attractive in biochemical sensing applications. Some deposition methods for the shell formation were developed to obtain a robust and clean crystal surface, which does not require a large volume of water or solvent, nor large energy and time. For example, high-quality few-layer molybdenum sulfide (MoS_2_) nanotubes with good morphology control were synthesized by combining the nanowire template with the CVD process [49]. As shown in Figure 3a, SiO_2_ NWs prepared and dispersed on silicon substrates are used as templates for depositing a thin film of MoS_2_. The cores are stable in CVD process and are etched away in HF acid solution. Moreover, the sheathes maintain complete tubular structures and the number of MoS_2_ layers can be reduced to four by shortening the growth time. Similarly, Zhang et al. prepared CdS nanotubes with tunable wall dimensions and optical microcavities through a simple thermal evaporation co-deposition technique using Sn nanowire templates [50].

On the other hand, the shell can be produced by chemical reactions [51]. Zhu et al. have developed a novel optimized process that can precisely control the inner diameter without involving dispersion, transfer and alignment steps, leading to robust, smooth and clean nanotubes [52]. As shown in the Figure 3b, Self-assembled SiO_2_ nanotubes are prepared from Si nanowires on a silicon-on-insulator (SOI) substrate through several oxidation and etching processes. The channel between the NTs and SOI substrates was open by dry etching, which is beneficial to the preparation of nanofluidic devices and biomedical applications. Furthermore, a single-phase tube with an accurate stoichiometric ratio is readily obtained. The chemical conversion of the template by gas- or vapor-phase avoids problems of the solid-phase reaction due to the reaction self-termination once the sacrificial template is exhausted. As an example, Sutter et al. converted sphalerite GaAs nanowires into single crystal cubic γ-Ga_2_S_3_ nanotubes by reacting with sulfur vapor [53]. The schematic reaction sequence is shown in Figure 3c, which involves the rapid diffusion of Ga ions through the growing sulfide shell. Ga_2_S_3_ is thicken and separated from the contracted GaAs core until the entire core is consumed. 

In recent years, the nanowire template approach has been developed to the fabrication of various devices with specific functions. For example, the ultra-fine Pt nanotubes with 1 nm wall thickness and several microns length has been synthesized, which enhances the oxygen reduction reaction performance [54]. Insulin fibers have been introduced as a biological template, which allows the diameter of Pd NWs be reduced from 9 nm to less than 5 nm. The full decomposition of the fibers provided the necessary smooth surface for Pt deposition. In addition, highly active FeN_x_ modified CNTs are synthesized using ZnO nanowire templates [55]. As shown in Figure 3d, ZnO nanowires undergo in situ polymerization of dopamine under FeCl_3_ environment and then pyrolyze in NH_3_. The evaporation of Zn created sufficient pores on the CNTs and lead to high aspect area, which can be useful for the applications of sensors, catalysis, fuel cells, and so on.

## 3. Device Mechanisms

The use of NTs and NWs provides a new platform for nanofluidic devices. Compared with planar semiconductor sensors, surface bonding with biochemical molecules affects the accumulation and depletion of carriers in the entire nanostructure, instead of just a small portion of it. This greatly improves the sensitivity and even realizes the possibility of detecting individual molecules, which is very attractive in chemical and bio-sensing. This chapter will give a brief overview focusing on the working mechanism and sensing principle of nanofluidic devices based on NWs and NTs.

### 3.1. Working Principle of Nanofluidic Cells Based on Nanowires/Nanotubes (NWs/NTs)

**Figure 4:**
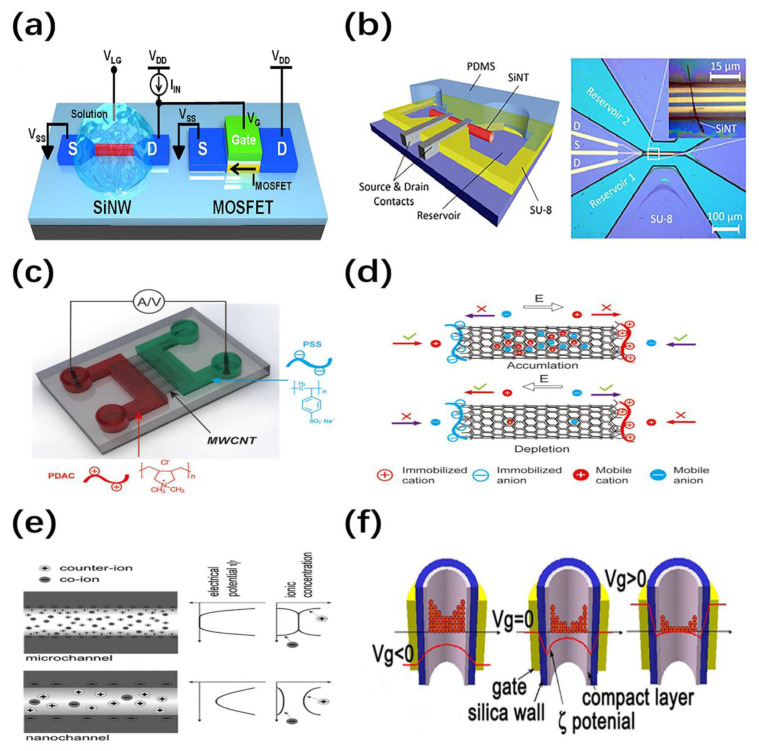
(**a**) Schematic illustration of a SiNW-based ion-sensitive field-effect transistor (ISFET) and a metal-oxide-semiconductor field-effect transistor (MOSFET) [57]; (**b**) schematic illustration of the SiNT-based ISFET device [61]; Schematic illustration of (**c**) the device structure and (**d**) working principle of a multi-walled carbon nanotubes (MWCNT) ionic diode filled with polyelectrolyte solutions (poly(diallyldimethylammonium chloride) (PSS) and poly(sodium 4-styrene-sulfonate) (PDAC)) [11]; (**e**) Schematic illustration of the ion environments and electric potential in microchannel and nanochannel [66]; (**f**) Schematic diagram of field-effect modulation of ion conductance in NT-based FETs [67].

The ion-sensitive field-effect transistor (ISFET) is one of the most promising devices in nanofluidic system for biochemical sensing application. The channel composed of NW and NT has a large specific surface area and high sensitivity, and can perform charge sensing and label-free detection of species in nanofluids [56,57]. We first describe the working mechanisms of NW/NT-based ISFETs to better understand the principle of biochemical sensing. As illustrated in Figure 4a, the basic structure of NW-based ISFET is similar to metal-oxide-semiconductor field-effect transistor (MOSFET) except the metal gate which is replaced by a liquid-sensing region [57]. The liquid gate voltage is applied to the electrolyte via the reference electrode, which influences the change of carriers and the conductance of devices [58]. In addition, the specific or non-specific binding of charged molecules can also generate the electric field making it equivalent to the role of a gate. As the diameter of the NWs decreases, the surface interactions cause more significant electrochemistry changes in the NWs. After the functionalization of NWs, it is possible to detect the pH value (H^+^ or OH^−^ ions) or selectively respond to the biochemical species (DNA, protein, gas, etc.) in nanofluid system [59]. On the other hand, nanotubes can not only provide the same function of sensing, but also serve as nanochannels to allow samples to flow through the interior. Due to the unique geometrical and electrical properties of NTs, it is extremely interesting to study the liquid and ion transport, as well as electrochemical detection of small molecules in nanofluids [60]. Hibst et al. integrated Si NTs into nanofluidic transistors in order to measure the ion diffusion of NaCl solution through NT, indicating that the fluid transmission in the nanoscale system can be simply considered by adjusting the diffusion coefficient [61]. As shown in Figure 4b, the NT-based ISFET consists of S/D electrical contact, fluidic supply and passivation layer covering (PDMS), in which the both ends of the Si NT are opened to connect two reservoirs.

However, the transport of water, ions and molecules through nanochannels is uniquely affected by special physical phenomena, including effects like electric double layer (EDL) and ion current rectification (ICR) [62]. ICR means that the ion current in one direction is greater than the other direction, and is determined by many factors, such as ion concentration, pH, surface charge density, curvature of channels, field effect modulation, etc. [63]. This asymmetric ion current is found in nanochannels and used to manufacture novel nanofluidic devices [64]. The control of ion transport rectification is usually achieved by functionalizing charged groups on the terminal or surface [65]. Compared with membrane-based nanopores, NTs with a better charge modification effect are more favorable nanochannels to increase the current rectification ratio. Take ion diode as an example, and the device structure and working mechanism are shown in Figure 4c,d [11]. Horizontally aligned nanotubes are employed as ion nanochannels, connecting two microchannels filled with nanofluidic. The mobile ions in the solution are driven by the voltage applied to both ends. ICR effect in the ion diode is caused by the accumulation or depletion of ion carriers. The oppositely charged polyelectrolytes are immobilized at the two terminals via strong physical absorption. The mobile cation and anion under the forward bias accumulate in the nanochannel by the electrostatic repulsion of both ends. In contrast, when a reverse bias is applied, the mobile ions are exhausted due to being attracted by the terminals. The increase and decrease of the overall conductance results in the on and off states of the ion diode. It should be noted that the charge carriers here are the ions in nanofluidic, rather than electrons or holes.

In particular, a unipolar ion environment could appear in nanotubes [48]. The general mechanism is shown in Figure 4e, where the surface of the nanotube is modified by charged groups, and the charged ions in the fluidic are attracted or repelled by the surface charges [66]. In the microchannel, EDL is much smaller than the diameter and the electrostatic potential decays from the surface potential to zero. However, the size of one dimension in the nanochannel is smaller than the Debye length, which enhances the concentrated distribution of counter ions and non-zero central potential. Therefore, electrostatic control of ions or molecules can be achieved in the entire nanotube. Similar to MOSFETs, nanofluidic FETs based on nanotubes exhibit unipolar ion transport. The gate voltage is employed to control the ion concentration by adjusting the surface potential or charge. In addition, the channel doping level can be determined by the intrinsic surface charge. Fan et al. implemented NT-based FETs for the first time, which exhibited fast field-effect modulation of ion conductance [67]. Figure 4f shows the schematic diagram, in which the surface of SiO_2_ is usually negatively charged due to the presence of hydroxyl and SiO^−^, thus the cations (k^+^) in nanofluidic serve as the majority carriers. The positive and negative gate voltages cause the depletion and accumulation of the cations, respectively, leading to the *p*-type behavior.

The implementation of devices based on NWs and NTs suggests the feasibility of using electric field to control the transport of charged species in nanofluidic systems. Subsequent work can focus on the fabrication of nanosensors through the surface functionalization with probe molecules, which can specifically recognize chemical or biological molecular targets.

### 3.2. Sensing Mechanism of Biochemical Sensors Based on NW/NT Nanofluidic Cells

With the development of micro-nano technology, sensors have made great progress in terms of size, cost, sensitivity and speed by using novel nanomaterials and new mechanisms. Nanowires, nanotubes and other microarrays are served as sensor elements to fix target molecules on the surface or manipulate their transmission, which has been widely used in nanofluidic platforms. Due to the small diameter and large aspect ratio, nanofluidic transistors based on NWs or NTs can even achieve molecular sensing at extremely low concentrations with various detection mechanisms. Generally, the sensor response depends on changes in mechanical and electrical signals, as well as the charge of nanostructures and biomolecules caused by the surface adsorption, reaction or transport of target molecules. There are several basic principles involved during the sensing processes. For example, the electric field can be changed by the surface polarization effect during the molecular adsorption, and the reaction products produced by the enzyme and the substrate also influence it.

**Figure 5:**
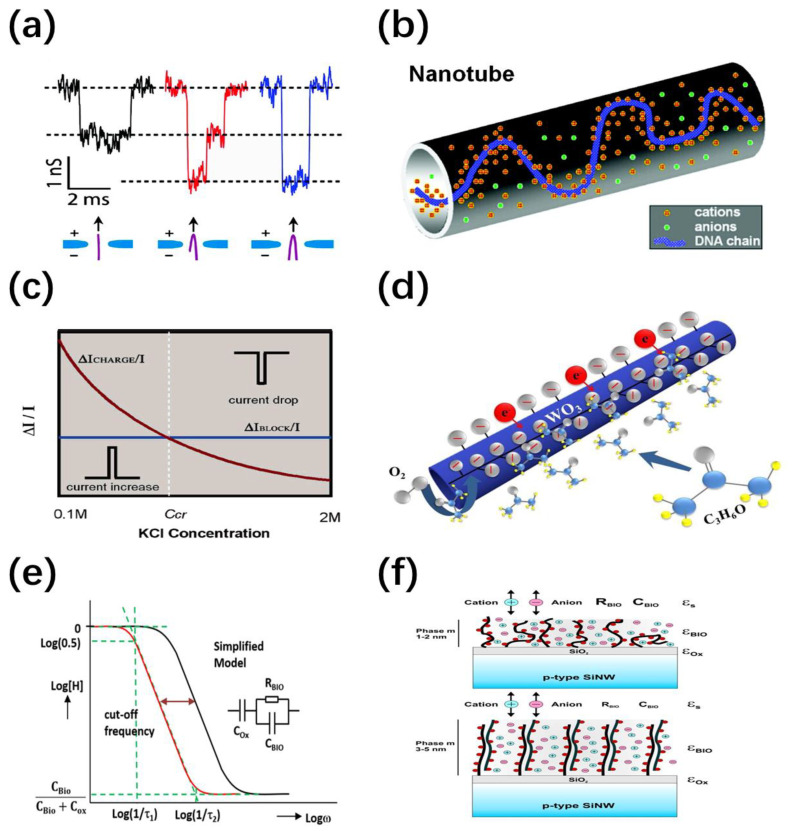
(**a**) Ion current response to the translocation of non-folded (black), partially folded (red), and fully folded (blue) DNA through a graphene nanopore [69]; (**b**) schematic illustration of the ionic distribution of counterions and co-ions in a nanotube with a DNA strand [70]; (**c**) schematic illustration of the current responses under different ion concentrations due to charge effect and blockade effect [70]; (**d**) schematic illustration of h-WO_3_ nanotubes detecting acetone gas [77]; (**e**) the transistor transfer function (TTF) spectrums of the original (**left**) and after biomolecule combined (**right**) devices. The inset shows the simplified model of the input impedance of the device, which can be described as an Resistor-Capacitance element (R_Bio_ and C_Bio_) of biomolecule layer in series with the gate oxide capacitance C_Ox_ [59]; (**f**) Schematic illustration of the attachment layers of single-stranded and double-stranded DNA molecules on the SiNW. The single-stranded DNA layer leads to a thin interface layer, where the negative charge introduces a certain surface charge density and forms an additional potential for ion diffusion. The charge composition of double-stranded DNA changes, resulting in a thicker layer, which allows ions more effectively diffuse to the oxide surface. The thickness of DNA layer, the diffusion of ions, the amount of DNA charges, and eventual changes in the dielectric constant ε_BIO_ will influence the biomolecule layer impedance, which allows for the expectation of the R_Bio_ and C_Bio_ values [59].

Electrical detection methods are usually used to reflect the concentration or translocation of chemical/biological molecules by monitoring the change of ion current in the nanochannel [68]. Nanopores have been used in single-molecule sensing for decades based on resistive pulse sensing mechanism. As shown in Figure 5a, when the particles pass through nanopores with comparable diameters, the ion current decreases transiently due to the blocking effect, which shows a broad prospect of rapid genome sequencing [69]. However, nanotubes with longer nanochannels and higher aspect ratios lead to a large number of blockade events and the interaction is more complicated, which also allows more obvious transient signals on the time scale. Fan et al. proposed an interaction model of charge effect and geometric effect to qualitatively explain the translocation of DNA molecules through nanotubes under different ion concentrations [70]. As shown in Figure 5b,c, charged molecules attract counter ions while repelling a portion of the volume of conductive ions. When the electrolyte concentration is lower than the critical concentration, the charge effect is stronger and will introduce more ion carriers. On the contrary, the volume effect is dominant and the ion conductance decreases. The ionic current encodes more biomolecule information such as charge, geometric characteristics and the evolution of DNA strand conformation through pulse duration, pulse amplitude and current attenuation characteristics, etc.

On the other hand, the nanostructure/electrolyte interface can be very sensitive to almost any specific surface reactions due to its large specific surface area which can provide more active centers. An individual molecule may induce significant changes in electronic properties in the following possible ways: (1) electrostatic gate control; (2) charge transfer; (3) charge scattering potential; (4) Schottky contact modulation [71,72]. For example, in oxygen gas sensing, the coverage of oxygen extracts electrons from the channel to form negatively charged oxygen ions (O^2−^, O^−^ and O_2_^−^), which results in a change in the width of the charge depletion layer. The presence of reducing gases and oxidizing gases determines the desorption and adsorption of oxygen, respectively. Therefore, the changes in resistance and conductivity of a NW can be manifested [73,74,75,76]. The sensing mechanism of the acetone sensor is demonstrated in Figure 5d [77]. Acetone or ethanol react with surface oxygen species and release electrons back into the conduction band of the nanostructure based on the following equations:

O_2_(g) → O_2_(ads), (1)

O_2_(ads) + e^−^ → O_2_^−^(ads), (2)

O_2_^−^ (ads) + e^−^ → 2O^−^ (ads), (3)

C_3_H_6_O(g) + 8O^−^ (ads) → 3CO_2_ + 3H_2_O + 8e^−^, (4)

C_2_H_5_O + 6O^−^ (ads) → 2CO_2_ + 3H_2_O(g) + 6e^−^, (5)

as a biosensor, the key point is the functionalization through the attachment of identification elements, which can be coupled with target analytes. The specific trapping of charged molecules is similar to the effect of applying a gate voltage, resulting in the accumulation and depletion of carriers. This was first applied in PH sensing that the conductance increased linearly with the pH value based on the mechanism of surface charge changes in the protonation and deprotonation procedures [78]. Biomolecules such as DNA, proteins, viruses, drugs, and neuronal signals have also been detected. Amorim reported that the cyclodextrin from biological enzyme products with hydroxyl groups tends to bind to the surface of SnO_2_ nanowires, changing its electrical conductivity [79]. The nanowire biosensor exhibits different current responses under various cyclodextrin concentrations.

It is worth mentioning that various factors will affect the surface properties of nanostructures. As a result, complex working mechanisms require precise models and schemes for calibration. The research on Stern and diffusion is of great importance to improve the accuracy of target molecule detection. A method of experimentally extracting the Stern layer capacitance (C_stern_) of Si NWs ISFET has been proposed which improves the development of ISFET-based biosensors [80]. So far, the commonly used detection mechanism is based on the drift of threshold voltage (V_TH_) or the change of NWs conductance when binding target biomolecules. However, there is a limitation of potential sensing by the Debye screening of counter ions in the test solution. Schwartz et al. developed a novel impedance sensing approach for Si NWs ISFET devices for DNA detection [59]. This approach based on the transistor transfer function (TTF) has potential to achieve effective biosensing in physiological buffers. As shown in Figure 5e, the black and red curves show the TTFs of the original device and the device after the biomolecule combination, respectively, in which the estimated concentration of biomolecules can be calculated from the difference in cutoff frequencies. The inset of Figure 5e is a simplified input impedance model composed of a biomolecular layer and an oxide layer. The molecules are immobilized on the surface of the nanowire, which changes the input impedance and frequency dependence of the device. In Figure 5f, the single-stranded DNA layer on the surface of the Si NW has introduced certain surface charges. After DNA hybridization, the thickness of the biomolecule layer and the amount of DNA charge as well as the ion diffusion barrier have all changed, resulting in different input impedances (R_BIO_ and C_BIO_).

On the other hand, NWs/NTs with enzyme-like activity is also a new type of nanozyme materials, which plays an important role in electrochemical biosensors for environmental and health monitoring [81]. Since the discovery of the peroxidase-like activity of Fe_3_O_4_ NPs, many research groups have devoted to the study of nanozymes [82]. NWs/NTs-based nanozyme immunoassay mainly performs three functions: target capture, magnetic separation, and color determination. In general, nanozymes catalyze the oxidation of a variety of chromogenic substrates in the presence of H_2_O_2_ to produce color reactions. This concept has been proven to detect H_2_O_2_ and other biomolecules such as glucose and lactic acid.

From the above analysis and reported work, the nanofluidic devices based on NTs and NWs are promising for the detection and sensing of chemical and biological species due to its real-time, label-free and ultra-high sensitivity advantages.

## 4. Device Applications for Chemical and Bio-Sensing

### 4.1. Chemical Sensing

#### 4.1.1. Ion Sensing

**Figure 6:**
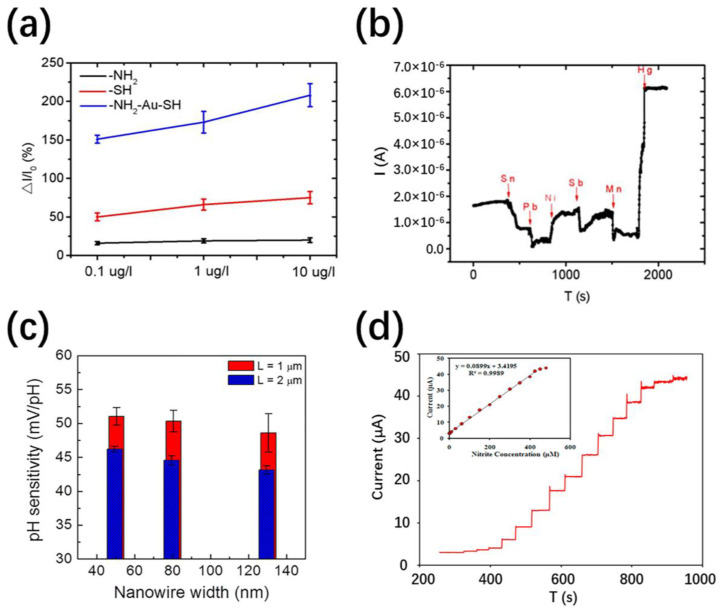
(**a**) △I/I_0_ as a function of mercury ion concentration for different modified SiNW devices [83]; (**b**) real-time current response to various metal ions of 10 μg/L^−1^ [83]; (**c**) the pH sensitivity of nanowires with different widths and gate lengths (L) of Effects of 1 and 2 μm [85]; (**d**) the current response of the Ag/halloysite nanotube (HNT)/MoS_2_-carbon paste electrode (CPE) to the nitrite with various concentrations. The inset shows the calibration curve of current versus nitrite concentration from 2 to 425 µM [86].

Nanotubes have been proven both theoretically and experimentally to be effective nanofluidic channels, which can work at the molecular level for PH and ion sensing. A gold nanoparticle-modified SiNWs array-based sensor for highly sensitive and selectivity detection of mercury ion has been reported (Figure 6a,b), which plays a potential role in monitoring the quality of water [83]. Cao et al. have demonstrated the electrical response of single-walled carbon (SWCNT) to the concentration, pH and ion type of the ionic solution when exposed to electrolyte [84]. The presence of K^+^ cations at low concentration inhibits the interaction of water with the inner surface of the CNT, thereby improving the gate control efficiency; while the higher ion concentration has a shielding effect on the gate voltage. In addition, the dependence of SWCNT with open ends on pH is opposite to that of unopened NTs or NWs, indicating their different pH sensing mechanisms.

The widths and lengths of Si NWs also have an influence on pH sensing [58,85]. As shown in Figure 6c, the NWs with smaller width and longer length exhibit higher pH sensitivities. This can be understood by the overlap of the electric field concentration area in the narrow channels and the small ratio between the source and drain electrode resistance and the total resistance in the long channels. More recently, Ghanei-Motlagh et al. demonstrated a new type of electrochemical sensor based on silver/halloysite nanotube/MoS_2_ nanocomposite [86]. This shows a relatively fast response time, low detection limit and good anti-interference performance for nitrite sensing at low concentration levels. A linear amperometry response to the concentration of nitrite from 2 to 425 µM is presented in Figure 6d. Nanostructured materials and their composite materials are considered promising candidates in the field of electrochemical sensing.

#### 4.1.2. Gas Sensing

**Figure 7:**
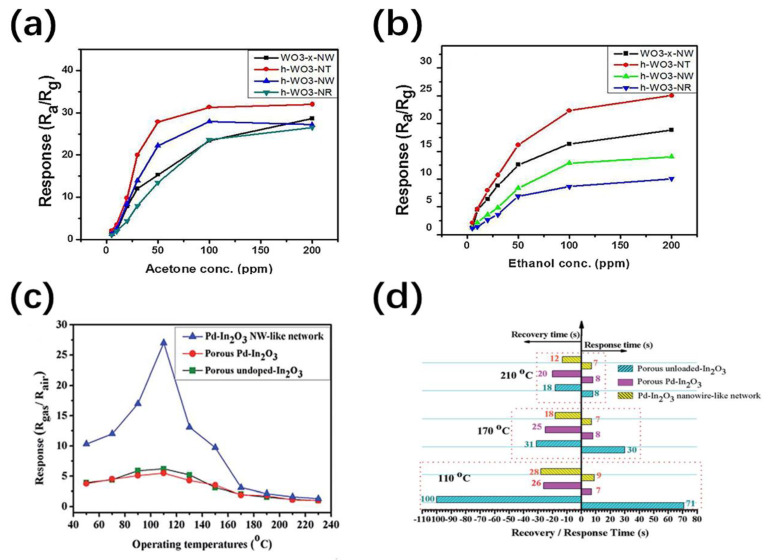
The response (R_a_/R_g_) of WO_3−x_ nanowire, h-WO_3_ nanotube, h-WO_3_ nanowire and h-WO_3_ nanorod versus acetone (**a**) and ethanol (**b**) concentration [77]; (**c**) the response of porous unloaded-In_2_O_3_, porous Pd–In_2_O_3_ and Pd–In_2_O_3_ NW-like network under different temperatures to 5 ppm NO_2_ [90]; (**d**) the response and recovery times of three samples to 5 ppm NO_2_ at 110, 170 and 210 °C [90].

Sensing and monitoring of various toxic gases such as H_2_S, CO, NO_2_, acetone, ethanol, etc. have become increasingly important in human life. NTs or NWs as sensing elements have great potential in high-precision gas sensing with high sensitivity, fast response, and good selectivity. As illustrated in Table 1, WO_3_ nanostructures have proved to be efficient materials for ethanol detection [87]. Different structural morphologies such as WO_3−x_ nanowires, h-WO_3_ nanowires, h-WO_3_ nanorods and h-WO_3_ nanotubes have also been prepared and tested [77]. As shown in Figure 7a,b, a relatively high sensitivity to acetone and ethanol have been observed in h-WO_3_ nanotubes (Ra/Rg = 32 and 26, respectively). This is due to the inherent local crystal defects and oxygen vacancies of obtained ultrathin-walled NTs are conducive to the adsorption of oxygen molecules and target gases, hence improving the sensing performance. Actually, it is difficult for gas molecules to penetrate into the interior of NTs due to the large aspect ratio. Therefore, porous nanotubes are proposed to break through the limitation of NTs in gas sensing. Compared with general SnO_2_ nanotubes, mesoporous SnO_2_ nanotubes exhibit an enhanced response sensitivity for methanol with fast response and recovery times of 3 s and 6 s, respectively [88]. In the same year, Choi et al. prepared various nanostructures (nonporous ZnO NTs, porous ZnO NTs, and porous ZnO film) by adjusting the sputtering power through polyvinyl acetate (PVA) nanowire template approach for hydrogen sensing [89]. Compared with the other two structures, the response of porous ZnO NTs to hydrogen sensing has been significantly enhanced. This is because both the inner and outer surfaces form a depletion layer and effectively sense gases simultaneously.

Noble metals and metal oxides have been demonstrated to modified the NWs or NTs to further improve the sensing performance. Pd-loaded In_2_O_3_ NW-like networks with excellent NO_2_ gas sensitivity was synthesized by Huang et al. using CNTs templates [90]. As shown in Figure 7c,d, Pd–In_2_O_3_ NW-like network sensors have significantly higher response (Ra/Rg = 27), shorter response and recovery times (9 s and 28 s, respectively) to 5 ppm NO_2_ at 110 °C. A synergy of electric and chemical effects is proposed to explain the influence of Pd loading. Choi et al. have experimentally coated Fe_2_O_3_ nanoparticles on the surface of SnO_2_ nanowires for ethanol sensing, which leads to the 1.48–7.54 times enhanced sensitivities of at 300 °C [91]. The conductivity of NWs mainly depends on the barrier height, which is increased with the number of grain boundaries. This multi-network structure and heterogeneous structure have introduced many crystal grains and grain boundaries, which contribute to achieve better sensing performance. Furthermore, composite materials, doping and heterostructures can also alleviate some difficulties in gas sensing [92,93]. Nevertheless, there are some existing problems such as low recovery, poor anti-interference, and insufficient sensitivity at room temperature, which need to be further developed in future gas-sensing technologies.

### 4.2. Bio-Sensing

#### 4.2.1. Molecular Detection

In addition to chemical species, various biomolecules can also be detected by specific binding with recognition groups on the surface of nanostructures. Proteins, drugs, hormones, viruses and so on are usually analyzed by the changes of electronic properties or ionic current after the formation of antigen–antibody complexes on immunosensors. The translocation time of small molecules through NTs is longer than that of nanopores and their interaction with the hydrophobic inner wall is stronger, so that the basic sensor signals are more obvious and can be easily captured.

**Figure 8:**
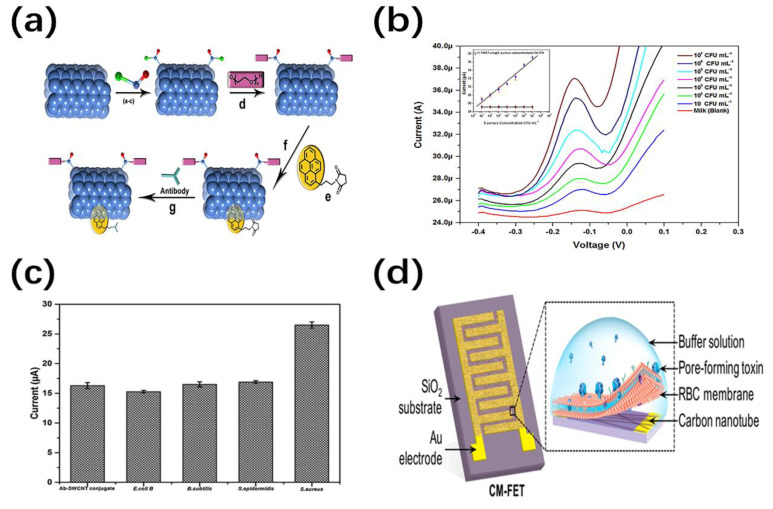
(**a**) Schematic illustration of the synthesis of modified MWCNT [94]; (**b**) the current versus voltage under different S. aureus concentrations from 10 to 10^7^ colony-forming units (CFU) mL^−1^ in milk sample [95]; (**c**) the Histogram of selectivity for S. aureus vs. E. coli B, B. subtilis, and S. epidermidis [95]; (**d**) schematic illustration of the cell-membrane-coated CNT field-effect transistor (FET) [96].

Recently, various research works have indicated that NTs/NWs-based nanofluidic devices are potential candidate for the detection of small biological molecules. Among them, the detection of pathogens plays an important role in the diagnosis and treatment of diseases. Multifunctional MWCNT-based magnetic fluorescent sensors have been fabricated by Liu et al., which shows high magnetic activity and fluorescence intensity linearly related to Vibrio alginolyticus (Va) concentration [94]. As shown in Figure 8a, after pretreatment of MWCNT, fluorescent reagent (Py) and antibody (Va-Ab) are used to recognize Va specifically, and non-specific adsorption of other strains was reduced by polyethylene glycol (PEG) modification. Moreover, electrochemical-based label-free sensing methods have been developed to improve the rapid, accurate and quantitative detection of pathogens. For example, Bhardwaj et al. demonstrated a paper-based immunosensor for the detection of foodborne virus [95]. In this work, Ab-SWCNT bio-conjugate simplifies the functionalization steps and improves the durability of the immunosensor. As shown in Figure 8b,c, these Ab-SWCNT immunosensors exhibit high linear behavior and high selectivity for S. aureus. Additionally, a novel biomimetic nanosensor was prepared for the quantitative detection of biotoxins using CNT FET coated with red blood cell membrane (Figure 8d) [96]. By responding to the change of the charge distribution on the FET surface during the toxin-biofilm interaction, the detection limit is reduced to fM range with accurate concentration dependence. As for other small biomolecules, a glutamate biosensor based on chitosan-CNT-AuNW composite material was developed by Kitikul et al. [97]. The device shows ideal sensing characteristics in sensitivity, linearity and detection limit, which is widely used in flow injection analysis. Furthermore, a construct of Fe_2_O_3_/CNTs hybrid nanozyme has been prepared by ALD for dopamine detection [98]. CNTs with good activity provide a dispersibility support for the ultra-small nanoparticles (<1 nm) with adjustable size. Compared with other alternative nanozymes, this work shows better sensitivity with a limit of detection as low as 0.11 μM. Li et al. has developed a novel Asp/Ce NT-based colorimetric nanosensor for cysteine (Cys) [99]. The devices show high sensitivity and selectivity for Cys in H_2_O_2_-free and enzyme-free conditions due to the intrinsic oxidase-like activity of Asp/Ce NTs. This detection method is quite simple, effective and highly repeatable by reducing the interference from the environment.

#### 4.2.2. DNA Sequencing

**Figure 9:**
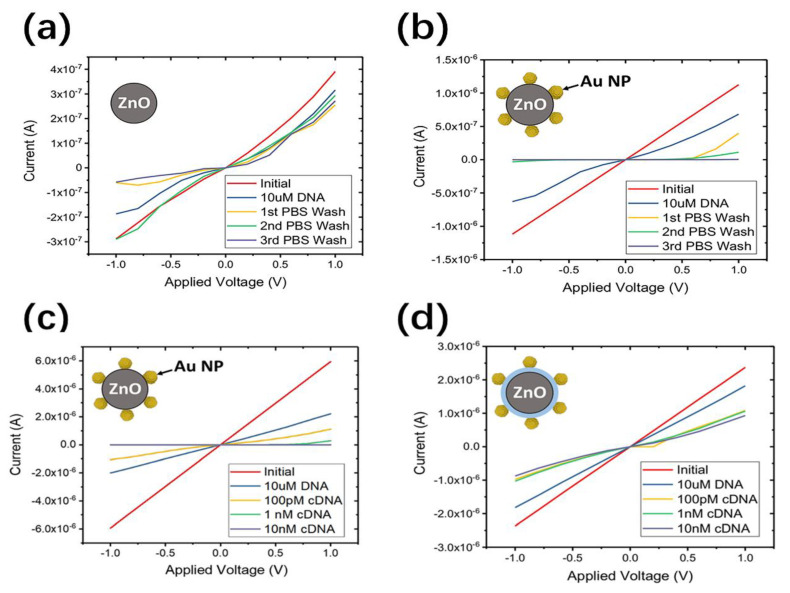
The response current-voltage curves of (**a**) bare, (**b**,**c**) Au NP decorated and (**c**) HfO_2_ Passivated ZnO NW sensors with phosphate bufered solution (PBS) solution or cDNA exposure. HfO_2_ passivation limits the change of NW conductivity with solution exposure (**c**), and AuNP modification maintains the sensitivity to DNA (**d**) [102].

**Figure 10:**
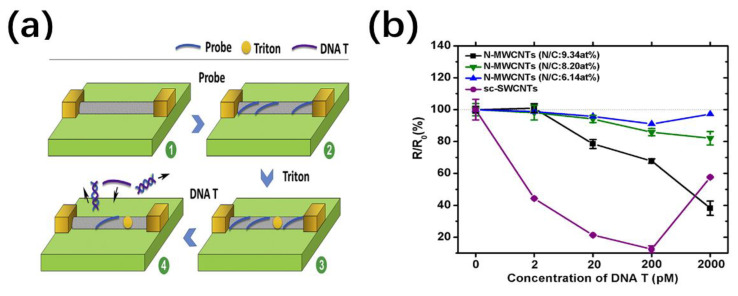
(**a**) Schematic illustration of the functionalization and sensing of the CNT-based biosensor [103]; (**b**) the response of N-doped MWCNTs with various N/C and sc-MWCNTs versus DNA concentration [103].

Well-designed NTs or NWs provide good opportunities for DNA real-time detection and sequencing [100]. It has been proven that single-stranded DNA can specifically and stably bind to the surface or the ends of functionalized NTs or NWs. Tahir et al. have been prepared the Zn-NPs loaded MWCNTs sensor with immobilized probe DNA strand [101]. During the hybridization process, the binding of a small amount of nucleic acid to the probe is converted into a directly measurable electrical signal. The electron doping of complementary DNA causes a great reduction in peak current, which shows three times higher selectivity compared to non-complementary DNA. It represents a simple and universal detection method of DNA sequencing with enhanced response characteristics. In another example, Danielson et al. first employed a magnetron-sputter gas aggregation method to functionalize ZnO NWs with Au NPs for DNA detection at sub-nanomolar concentrations [102]. As shown in Figure 9, the attachment of the negatively charged DNA aptamer causes the carrier depletion of the nanowire and transforms it into Schottky-like behavior. The initial introduction of complementary DNA hybridization shows high sensitivity with ~1 µA current drop at 100 pM. In addition, passivation treatment by depositing a HfO_2_ layer can alleviate the degradation of NWs exposed to aqueous solutions, which will produce a conductivity response similar to the negative charge signals. Recently, chemical resistance biosensors based on semiconductor SWCNTs (sc-SWCNTs) and nitrogen-doped MWCNTs (N-MWCNTs) have been compared for DNA sequencing derived from avian influenza virus H5N1 (Figure 10) [103]. The relatively higher sensitivity to the DNA target sequence was found in sc-SWCNTs with the lowest detection concentration down to 2 pm (20 pm in N-MWCNTs). This can be attributed to the Schottky barrier modulation effect at the CNT/metal interface, which exists only in semiconductor NTs but not in metallic NTs [104]. The amine groups on N-MW CNTs facilitate the adsorption of DNA probes while change the electronic properties of nanotubes.

However, nucleic acid detection requires high ionic strength solutions to achieve high density of negative charges, which results in an undesirable small Debye length. To begin with, the electrostatic adsorption of DNA probes parallel to the NW surface was employed to overcome the limitation of Debye screening effect [105]. Alternatively, the surface functionalization of neutrally charged peptide nucleic acid (PNA) for gene sensing has also been demonstrated. The PNA probe-modified CNT device developed by Fortunati has realized direct measurement of DNA from genetically modified (GM) Soy, reaching the threshold level of soybean meal verification [106]. The enhanced sensitivity of CNTs can be attributed to the intrinsic characteristics of PNA, which provides high recognition efficiency. On the other hand, the compatibility of NWs/NTs-based ISFET sensor arrays with Complementary Metal Oxide Semiconductor (CMOS) technology allows them to be integrated on a large scale. Xu et al. comprehensively optimized the sensitivity of CMOS-ISFET to biomolecule-DNA based on the device design of size and bias [107]. The experiments and model show that it is an effective way to enhance the charge detection signal of the device, by biasing at near-threshold, reducing the channel-to-sensing area ratio and minimizing the double-layer capacitance of the sensing electrode.

## 5. Conclusions

With the advancement of micro-nano technology, people have been increasingly interested in the basic research of nanofluid transport at the nanoscale. The NWs and NTs introduced here as 1D nanostructured materials have unique properties, which are very attractive for advanced preparation and optimization of novel nanofluidic cells for chemical and biological sensing, especially for small molecule detection and DNA sequencing. The robust and uniform 1D nanostructures prepared by the improved synthesis methods have controllable morphology, size, composition and easily functionalized surfaces, which provide a foundation for the fabrication of nanofluidic devices and sensing applications.

Additionally, nanotubes as channels and sensor elements have large specific surface areas and there are special physical transport phenomena at the nanometer scale, such as ICR and a unipolar ion environment. Compared with nanopores, better electrostatic tuning in time and space makes nanofluidic devices based on NWs and NTs powerful tools for environmental analysis and molecular sensing. Similar to planar semiconductor sensors, NWs/NTs-based devices have been improved in terms of size, sensitivity, speed and specificity, which enable the detection and manipulation of few charged species and even single molecules. Various detection mechanisms have been employed like static control, charge transfer and charge scattering, etc. Most of them rely on the basic signal changes of the nanostructure when the target molecule interacts with the surface receptor during the transfer process, which encodes molecular geometry, charge, concentration and other information through electronic or ionic current.

So far, there are still some limitations and challenges to be solved in NWs/NTs-based nanofluidic research for chemical and bio-sensing. To begin with, the main difficulty is the micro/nanofabrication technology in materials sorting and device fabrication, which limits the large-scale industrial production and applications. Moreover, high signal-to-noise ratio, low detection limit, and fast test analysis is another urgent problem to be solved. Hybrid detection mechanisms and accurate device models can be explored to reduce the noise and interference of functional devices. It is also possible to adopt novel devices structures and composite materials to improve response and selectivity. For example, incorporating multiple aligned or three-dimensional heterogeneous NWs/NTs into the devices may be an effective way. Further research should be conducted in material preparation, device fabrication and related applications. Nevertheless, the use of 1D nanostructure will deepen our understanding of the basic transport phenomena of nanofluids and open a new research paradigm in the areas of electronics, optoelectronics, chemistry and bio-sensing.

## Figures and Tables

**Table 1:** WO_3_ sensors for ethanol detection reported in literature [86].

Materials	Concentration(ppm)	Response
WO_3_	300	16.9
WO_3_ nanoplates	400	~12
WO_3_ hollow spheres	500	6.14
WO_3_ nanotube bundles	400	~38

## Data Availability

The data presented in this study are available in article.
